# Crystal structure of (μ-*N*-allyl­thio­urea-κ^2^ *S*:*S*)bis­[μ-bis­(di­phenyl­phosphanyl)methane-κ^2^ *P*:*P*′]bis­[bromido­copper(I)] aceto­nitrile disolvate

**DOI:** 10.1107/S2056989015015637

**Published:** 2015-08-26

**Authors:** Mareeya Hemman, Chaveng Pakawatchai, Jaursup Boonmak, Sujittra Youngme, Saowanit Saithong

**Affiliations:** a Department of Chemistry, Faculty of Science, Prince of Songkla University, Hat Yai, Songkhla, 90112, Thailand; b Department of Chemistry and Center of Excellence for Innovation in Chemistry, Faculty of Science, Prince of Songkla University, Hat Yai, Songkhla, 90112, Thailand; c Materials Chemistry Research Center, Department of Chemistry and Center of Excellence for Innovation in Chemistry, Faculty of Science, Khon Kaen University, Khon Kaen, 40002, Thailand

**Keywords:** crystal structure, copper(I) complex, di­phenyl­phosphino­methane, *N′*-allyl­thio­urea, hydrogen bonding, C—H⋯π inter­actions, π–π stacking inter­actions

## Abstract

The reaction of cuprous bromide with a mixture of 1,1-bis­(di­phenyl­phosphan­yl)methane (dppm: C_25_H_22_P_2_) and *N*-allyl­thio­urea (ATU: C_4_H_8_N_2_S) in aceto­nitrile yielded the title solvated dinuclear complex, [Cu_2_Br_2_(C_4_H_8_N_2_S)(C_25_H_22_P_2_)_2_]·2C_2_H_3_N or [Cu_2_Br_2_(ATU)(dppm)_2_]·2CH_3_CN. Both Cu^+^ ions adopt distorted tetra­hedral geometries, being coordinated by one terminal Br atom, one μ^2^-S atom of the bridging ATU ligand and two P atoms of the bridging dppm ligands. Within the complex, intra­molecular C—H⋯S, C—H⋯π, N—H⋯Br and π–π stacking inter­actions are observed. In the crystal, the components are linked by N—H⋯Br and C—H⋯N hydrogen bonds and weak π–π stacking inter­actions, generating chains propagating in the [100] direction.

## Abstract 2

In the solvated dinuclear complex [Cu_2_Br_2_(ATU)(dppm)_2_]·2CH_3_CN, both Cu^+^ ions adopt distorted tetra­hedral geometries, being coordinated by one terminal Br atom, one μ^2^-S atom of the bridging ATU ligand and two P atoms of the bridging dppm ligands. Within the complex, intra­molecular C—H⋯S, C—H⋯π, N—H⋯Br and π–π stacking inter­actions are observed. In the crystal, the components are linked by N—H⋯Br and C—H⋯N hydrogen bonds and weak π–π stacking inter­actions, generating chains propagating in the [100] direction.

## Chemical context   

Copper(I) complexes with mixed ligands containing diphosphine are of inter­est because of their attractive coordination chemistry and several potential applications resulting from their photophysical properties (Yam *et al.*, 1999 ▸; Jin *et al.*, 2009 ▸; Zhang *et al.*, 2011 ▸, 2014 ▸; Tsiaggali *et al.*, 2013 ▸), anti­bacterial activity and their inter­action ability with native calf thymus DNA (CT–DNA) (Tsiaggali *et al.*, 2013 ▸). One of the diphosphine ligands, 1,1-bis­(diphenylphosphino)methane 
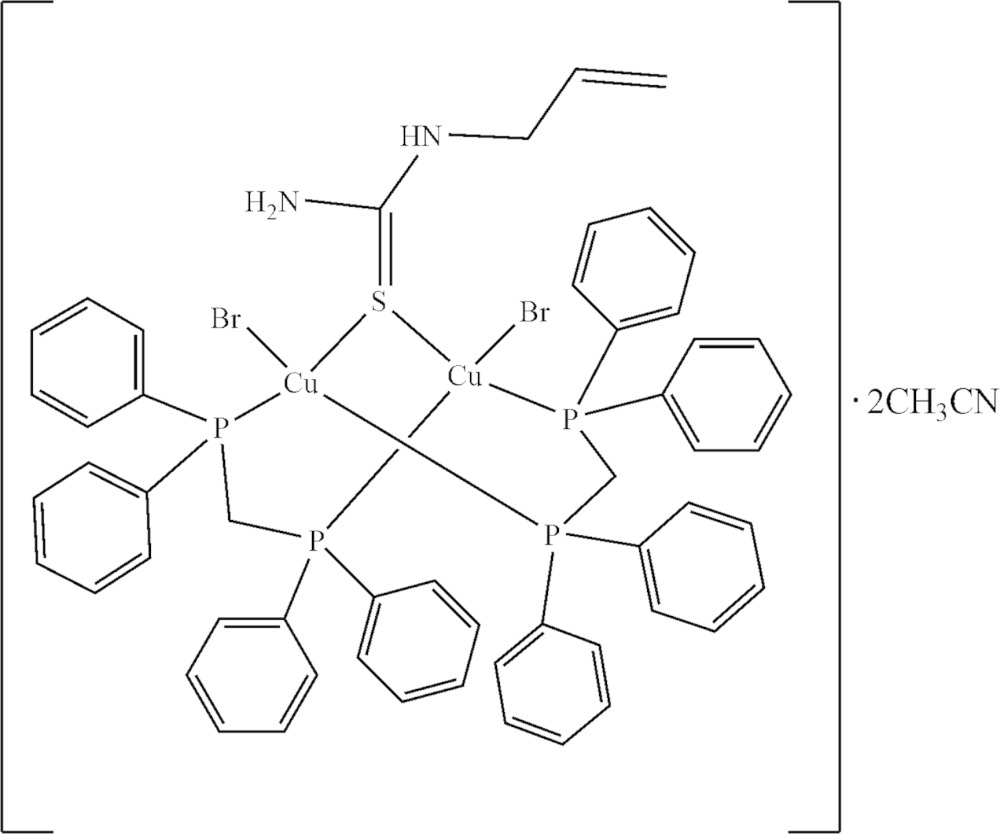
(dppm: C_25_H_22_P_2_) is a bridging bidentate ligand which is effective in forming various structures with many types of additional ligands, leading to copper(I) complexes as mononuclear, dinuclear, trinuclear and tetra­nuclear models, depending on the nature of the mixed-ligand partners and the stoichiometric ratio between the reactants and experimental conditions (Ruina *et al.*, 1997 ▸; Pérez-Lourido *et al.*,1998 ▸; Dennehy *et al.*, 2009 ▸; Zhang *et al.*, 2011 ▸). *N*-allyl­thio­urea (ATU: C_4_H_8_N_2_S) is a substituted thio­urea ligand, which contains sulfur donor atoms that can bind to a copper(I) ion *via* a variety of bonding modes (Filinchuk *et al.*, 1996 ▸, 2001 ▸; Olijnyk *et al.*, 2003 ▸). A family of allyl­thiuorea–metal complexes has been studied for their potential photonic applications and non-linear optical (NLO) efficiencies (Perumal & Babu, 2008 ▸, 2012 ▸). In this paper, we report the synthesis and structure of a mixed-ligand copper(I) complex with dppm and ATU ligands.

## Structural commentary   

**Figure 1:**
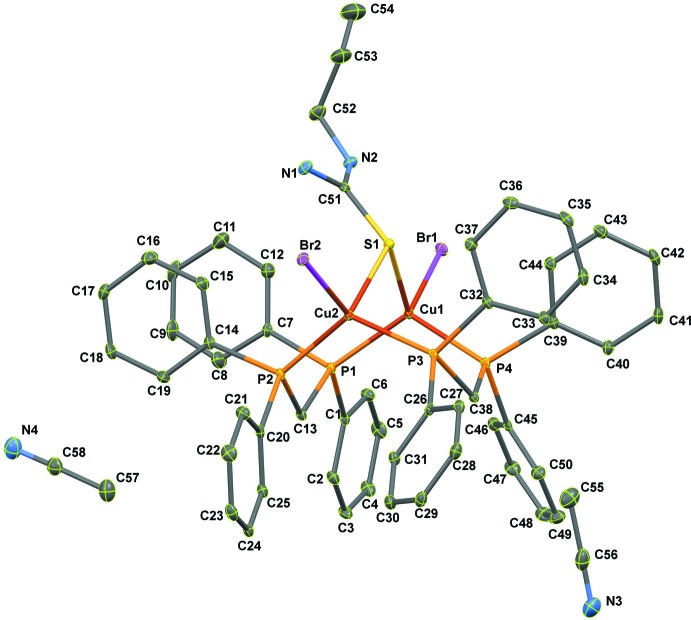
The mol­ecular structure of (I) showing 50% probability displacement ellipsoids.

The title complex [Cu_2_Br_2_(ATU)(dppm)_2_]·2CH_3_CN, (I), is shown in Fig. 1 ▸. The discrete neutral dinuclear complex contains an asymmetric triply bridged dicopper(I) core forming two six-membered rings in a chair conformation, Cu1—S1—Cu2—P1—C13—P2 and Cu1—S1—Cu2—P3—C38—P4, sharing the Cu1—S1—Cu2 part. Each Cu^I^ atom is coordinated by P atoms from the different dppm ligands, one *μ* ^2^-S bridging atom of ATU and the bromide ion as a terminal ligand, forming a distorted tetra­hedral environment, as illus­trated by the range of angles around the Cu atoms [100.87 (2)– 116.49 (3)° for Cu1 and 97.45 (3)–119.31 (3)° for Cu2]. Both Cu^I^ atoms share a tetra­hedral corner *via* an S-atom bridge.

The Cu⋯Cu distance in (I) is 3.3780 (7) Å, which is similar to the Cu⋯Cu distance of 3.375 (2) Å in a copper(I) complex containing an S–bridging ligand and bidentate bridging dppm, [Cu_2_I_2_(C_3_H_8_N_2_S)(C_25_H_22_P_2_)_2_]·1.5CH_3_CN (Nimthong *et al.*, 2013 ▸) and much longer than 2.8 Å which is the sum of the van der Waals radii of the copper atoms (Yam *et al.*, 2001 ▸). The Cu—P bond lengths in (I) (Table 1 ▸) range from 2.2604 (8) to 2.2803 (8) Å and the Cu—S bond lengths of the bridges, 2.3543 (8) and 2.3520 (8) Å, are likewise similar to the those in the above mentioned complex [Cu—P = 2.2563 (10)–2.2786 (11) Å; Cu—S = 2.3450 (11) and 2.3493 (11) Å]. The Cu1—Br1 and Cu2—Br2 bond lengths of 2.5126 (4) and 2.5238 (4) Å, respectively, are slightly longer than the Cu—Br(terminal) [2.4826 (5) Å] for [CuBr(PPh_3_)_3_]·CH_3_CN (Altaf & Stoeckli-Evans, 2010 ▸)

**Figure 2:**
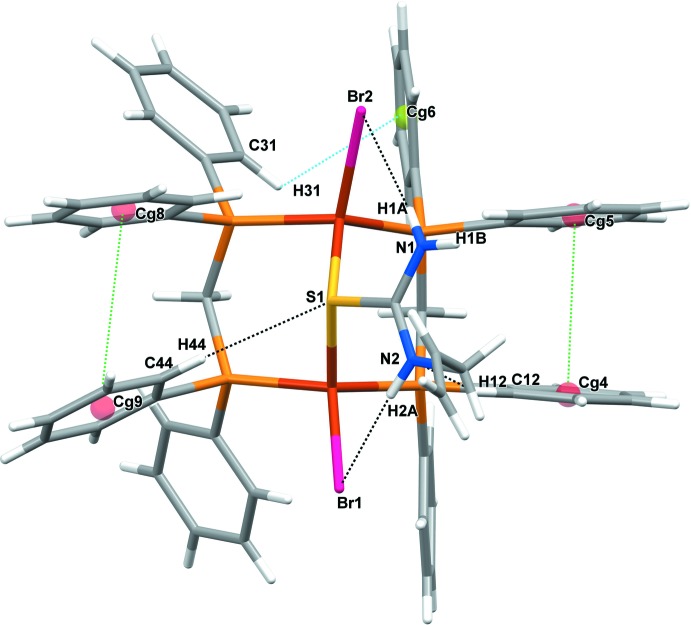
Intra­molecular inter­actions in (I)

The intra­molecular inter­action C44—H44⋯S1 (Table 2 ▸) has an effect on the Cu1–S1–Cu2 plane, bending it from the Cu_2_P_2_ plane of the Cu1–S1–Cu2–P3–C38–P4 chair conformation by an angle [72.59 (3)°] that is significantly larger than that for the Cu1–S1–Cu2–P1–C13–P2 plane [45.68 (2)°]. Thus, the C—H⋯S effect might be the influence that leads to the longer distance between the centroids (*Cg*8: C39–C44, *Cg*9: C45–C50; *Cg*4: C7–C12 and *Cg*5: C13–C18) of the phenyl rings, *Cg*8⋯*Cg*9 [4.0356 (17) Å] compared with the *Cg*4⋯*Cg*5 distance [3.6097 (19) Å]. The intra­molecular C—H⋯π inter­action (C31—H31⋯*Cg6*) also imposes a contact, of 3.476 (3) Å, between the C*sp* ^2^ atom of the phenyl ring and another phenyl ring centroid (C20–C25) of the other dppm ligand. An inter­action (C12—H12⋯N2) between *Cg5* and the NH_2_ group is also observed. In addition, two intra­molecular N—H⋯Br inter­actions are found between the NH_2_ group of ATU and Br atoms. A perspective view of all the intra­molecular inter­actions in (I) is depicted in Fig. 2 ▸.

## Supra­molecular features   

**Figure 3:**
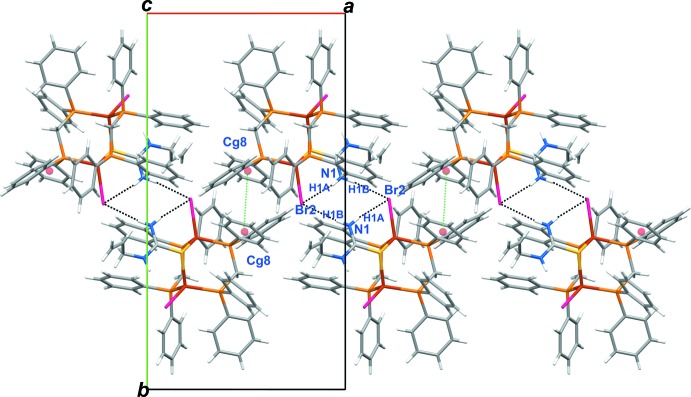
A chain in the structure of (I) mediated by N—H⋯Br hydrogen bonds and aromatic π–π stacking.

In the crystal, neighbouring dinuclear mol­ecules form a hydrogen-bonded dimer held together by two N—H⋯Br bonds as a cyclic pattern with its symmetry–equivalent partner, generated by a crystallographic inversion center (symmetry code: −*x*, 2 − *y*, −*z*), which generates 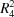
(8) loops. Moreover, the dimers are linked together by very weak π–π stacking of the *Cg*8⋯*Cg*8^iii^ rings [3.9338 (16) Å, symmetry code: (iii) 1 − *x*, 2 − *y*, −*z*] generating a chain of alternating N—H⋯Br links and π–π stacking running along [100], as shown in Fig. 3 ▸. The N atoms of the aceto­nitrile solvent mol­ecules both accept C—H⋯N inter­actions (C55—H55⋯N4^ii^ and C50—H50⋯N3^i^; symmetry codes: (i) −*x* + 1, *y* + 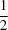, −*z* + 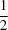; (ii) = −*x*, *y* − 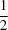, −*z* + 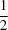) from the dppm phenyl rings. Numerical details of the hydrogen–bond geometry are given in Table 2 ▸.

## Database survey   

A search of the Cambridge Structural Database (Version 5.36, update November 2014; Groom & Allen, 2014 ▸) found 309 complexes of copper(I) with mixed dppm and other ligands. There are six copper(I) complexes with an ATU ligand, three complexes containing only an ATU ligand and three complexes containing a mixed ATU and other ligands. However, there is only one structure that has a similar core structure and coordination mode to the title compound, [Cu_2_I_2_(C_3_H_8_N_2_S)(dppm)_2_]·1.5CH_3_CN, studied by Nimthong *et al.* (2013 ▸).

## Synthesis and crystallization   

Copper(I) bromide (0.07 g, 0.49 mmol) was added to a solution of 1,1-bis­(di­phenyl­phosphino)methane (0.19 g, 0.50 mmol) in 30 ml aceto­nitrile at 338 K. The mixture was refluxed for two h and a white precipitate was formed. After that, *N*-allyl­thio­urea (0.06 g, 0.52 mmol) was added and further refluxed for four h and the precipitate slowly disappeared. Colorless crystals were obtained after the clear solution was left to evaporate at room temperature for a few days. The yield was 33% based on CuBr. Calculated for C_58_H_58_Br_2_Cu_2_N_4_P_4_S: C 55.55, H 4.66, N 4.47 and S 2.56%. Found: C 55.28, H 4.28, N 3.68 and S 2.54%. The main IR bands (KBr disc, cm^−1^): 3282 [ν(NH_2_)], 3168 [ν(NH_2_)], 3058 [ν(=C—H)_ph_], 1580 [ν(C=S)] and 1110 [ν(P—C_ph_)].

## Refinement   

Crystal data, data collection and structure refinement details are summarized in Table 3 ▸. Hydrogen atoms bonded to carbon were included at geometrically idealized positions and refined as riding with *U*_iso_(H) = 1.2*U*_eq_(C) or 1.5*U*_eq_(C_methyl_). The N—H atoms were located in a difference map and the coordinates were refined with an N—H distance restraint of 0.86 Å and with *U*_iso_(H) = 1.2*U_eq_*(N). 

## Supplementary Material

Crystal structure: contains datablock(s) I. DOI: 10.1107/S2056989015015637/hb7479sup1.cif

Structure factors: contains datablock(s) I. DOI: 10.1107/S2056989015015637/hb7479Isup2.hkl

CCDC reference: 1419827

Additional supporting information:  crystallographic information; 3D view; checkCIF report

## Figures and Tables

**Table 1:** Selected bond lengths ()

Cu1P1	2.2795(8)	Cu2P2	2.2604(8)
Cu1P4	2.2803(8)	Cu2P3	2.2730(8)
Cu1S1	2.3543(8)	Cu2S1	2.3520(8)
Cu1Br1	2.5126(4)	Cu2Br2	2.5238(4)

**Table 2:** Hydrogen-bond geometry (, ) *Cg*6 is the centroid of the C20C25 ring.

*D*H*A*	*D*H	H*A*	*D* *A*	*D*H*A*
C12H12N2	0.95	2.56	3.438(4)	154
C44H44S1	0.95	2.87	3.751(3)	154
C50H50N3^i^	0.95	2.57	3.462(5)	156
C55H55*C*N4^ii^	0.98	2.57	3.338(5)	136
N1H1*A*Br2	0.84(2)	2.74(2)	3.580(3)	172(4)
N1H1*B*Br2^iii^	0.85(2)	2.61(2)	3.415(3)	158(3)
N2H2*A*Br1	0.85(2)	2.63(2)	3.468(3)	166(3)
C31H31*Cg*6	0.95	2.97	3.476(3)	115

Symmetry codes: (i) 
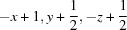
; (ii) 
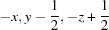
; (iii) 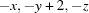.

**Table 3:** Experimental details

Crystal data	
Chemical formula	[Cu_2_Br_2_(C_4_H_8_N_2_S)(C_25_H_22_P_2_)_2_]2C_2_H_3_N
*M*_r_	1253.92
Crystal system, space group	Monoclinic, *P*2_1_/*c*
Temperature (K)	100
*a*, *b*, *c* ()	13.7569(7), 24.2730(11), 17.8527(8)
()	111.317(2)
*V* (^3^)	5553.5(5)
*Z*	4
Radiation type	Mo *K*
(mm^1^)	2.40
Crystal size (mm)	0.47 0.30 0.13

Data collection	
Diffractometer	Bruker APEXII CCD
Absorption correction	Multi-scan (*SADABS*; Bruker, 2013 ▸)
*T*_min_, *T*_max_	0.489, 0.745
No. of measured, independent and observed [*I* > 2(*I*)] reflections	70511, 10194, 8464
*R*_int_	0.064
(sin /)_max_ (^1^)	0.603

Refinement	
*R*[*F* ^2^ > 2(*F* ^2^)], *wR*(*F* ^2^), *S*	0.032, 0.067, 1.04
No. of reflections	10194
No. of parameters	651
No. of restraints	3
H-atom treatment	H atoms treated by a mixture of independent and constrained refinement
_max_, _min_ (e ^3^)	0.68, 0.39

Computer programs: *APEX2* and *SAINT* (Bruker, 2013 ▸), *SHELXS97* (Sheldrick, 2008 ▸), *SHELXL2014*/7 (Sheldrick, 2015 ▸), *Mercury* (Macrae *et al.*, 2008 ▸) and *WinGX* publication routines (Farrugia, 2012 ▸).

## supplementary crystallographic information

### Crystal data

**Table:**

[Cu_2_Br_2_(C_4_H_8_N_2_S)(C_25_H_22_P_2_)_2_]·2C_2_H_3_N	*F*(000) = 2552
*M**_r_* = 1253.92	*D*_x_ = 1.500 Mg m^−^^3^
Monoclinic, *P*2_1_/*c*	Mo *K*α radiation, λ = 0.71073 Å
*a* = 13.7569 (7) Å	Cell parameters from 9875 reflections
*b* = 24.2730 (11) Å	θ = 2.9–25.3°
*c* = 17.8527 (8) Å	µ = 2.40 mm^−^^1^
β = 111.317 (2)°	*T* = 100 K
*V* = 5553.5 (5) Å^3^	Block, colourless
*Z* = 4	0.47 × 0.30 × 0.13 mm

### Data collection

**Table:**

Bruker APEXII CCD diffractometer	8464 reflections with *I* > 2σ(*I*)
φ and ω scans	*R*_int_ = 0.064
Absorption correction: multi-scan (*SADABS*; Bruker, 2013)	θ_max_ = 25.4°, θ_min_ = 2.9°
*T*_min_ = 0.489, *T*_max_ = 0.745	*h* = −16→16
70511 measured reflections	*k* = −29→29
10194 independent reflections	*l* = −21→21

### Refinement

**Table:**

Refinement on *F*^2^	Primary atom site location: structure-invariant direct methods
Least-squares matrix: full	Secondary atom site location: difference Fourier map
*R*[*F*^2^ > 2σ(*F*^2^)] = 0.032	Hydrogen site location: inferred from neighbouring sites
*wR*(*F*^2^) = 0.067	H atoms treated by a mixture of independent and constrained refinement
*S* = 1.04	*w* = 1/[σ^2^(*F*_o_^2^) + (0.0189*P*)^2^ + 10.1133*P*] where *P* = (*F*_o_^2^ + 2*F*_c_^2^)/3
10194 reflections	(Δ/σ)_max_ = 0.003
651 parameters	Δρ_max_ = 0.68 e Å^−^^3^
3 restraints	Δρ_min_ = −0.39 e Å^−^^3^

### Special details

**Table:**

Geometry. All e.s.d.'s (except the e.s.d. in the dihedral angle between two l.s. planes) are estimated using the full covariance matrix. The cell e.s.d.'s are taken into account individually in the estimation of e.s.d.'s in distances, angles and torsion angles; correlations between e.s.d.'s in cell parameters are only used when they are defined by crystal symmetry. An approximate (isotropic) treatment of cell e.s.d.'s is used for estimating e.s.d.'s involving l.s. planes.

### Fractional atomic coordinates and isotropic or equivalent isotropic displacement parameters (Å^2^)

**Table:**

	*x*	*y*	*z*	*U*_iso_*/*U*_eq_	
Cu1	0.19238 (3)	0.77461 (2)	0.04894 (2)	0.01110 (8)	
Cu2	0.24847 (3)	0.90361 (2)	0.12313 (2)	0.01111 (8)	
Br1	0.09590 (2)	0.71918 (2)	−0.07468 (2)	0.01574 (7)	
Br2	0.22027 (2)	1.00599 (2)	0.10111 (2)	0.01554 (7)	
S1	0.17431 (5)	0.86405 (3)	−0.00549 (4)	0.01173 (15)	
P1	0.11946 (6)	0.76195 (3)	0.14370 (4)	0.01021 (15)	
P2	0.18113 (6)	0.87778 (3)	0.21589 (4)	0.01031 (15)	
P3	0.41788 (6)	0.88387 (3)	0.14411 (4)	0.00975 (15)	
P4	0.36776 (6)	0.76019 (3)	0.08920 (4)	0.01024 (15)	
N1	0.0202 (2)	0.93599 (11)	−0.05759 (16)	0.0189 (6)	
N2	−0.01468 (19)	0.84941 (10)	−0.11461 (15)	0.0163 (5)	
C1	0.1247 (2)	0.69122 (11)	0.18132 (17)	0.0116 (6)	
C2	0.1656 (2)	0.67627 (12)	0.26213 (17)	0.0165 (6)	
H2	0.1930	0.7037	0.3022	0.020*	
C3	0.1666 (3)	0.62129 (12)	0.28439 (18)	0.0199 (7)	
H3	0.1960	0.6113	0.3396	0.024*	
C4	0.1255 (3)	0.58136 (12)	0.22736 (19)	0.0214 (7)	
H4	0.1262	0.5439	0.2430	0.026*	
C5	0.0828 (3)	0.59573 (12)	0.14673 (19)	0.0218 (7)	
H5	0.0532	0.5681	0.1072	0.026*	
C6	0.0831 (2)	0.65008 (12)	0.12364 (18)	0.0171 (6)	
H6	0.0550	0.6595	0.0682	0.021*	
C7	−0.0200 (2)	0.77548 (11)	0.11547 (17)	0.0128 (6)	
C8	−0.0716 (3)	0.76700 (16)	0.1687 (2)	0.0321 (9)	
H8	−0.0335	0.7551	0.2220	0.039*	
C9	−0.1775 (3)	0.77573 (16)	0.1446 (2)	0.0328 (9)	
H9	−0.2118	0.7699	0.1815	0.039*	
C10	−0.2336 (2)	0.79270 (13)	0.0679 (2)	0.0247 (8)	
H10	−0.3067	0.7984	0.0516	0.030*	
C11	−0.1844 (3)	0.80142 (15)	0.0147 (2)	0.0319 (8)	
H11	−0.2233	0.8132	−0.0386	0.038*	
C12	−0.0770 (3)	0.79299 (14)	0.03881 (19)	0.0241 (7)	
H12	−0.0429	0.7994	0.0019	0.029*	
C13	0.1803 (2)	0.80300 (11)	0.23586 (16)	0.0119 (6)	
H13A	0.2531	0.7903	0.2635	0.014*	
H13B	0.1421	0.7965	0.2725	0.014*	
C14	0.0483 (2)	0.89986 (11)	0.20261 (17)	0.0123 (6)	
C15	−0.0113 (2)	0.92542 (13)	0.13206 (18)	0.0209 (7)	
H15	0.0174	0.9320	0.0919	0.025*	
C16	−0.1132 (3)	0.94168 (14)	0.1190 (2)	0.0269 (8)	
H16	−0.1536	0.9593	0.0700	0.032*	
C17	−0.1553 (2)	0.93239 (12)	0.17676 (18)	0.0176 (7)	
H17	−0.2246	0.9439	0.1682	0.021*	
C18	−0.0967 (3)	0.90642 (14)	0.2469 (2)	0.0263 (8)	
H18	−0.1262	0.8994	0.2865	0.032*	
C19	0.0052 (3)	0.89033 (14)	0.26059 (19)	0.0245 (8)	
H19	0.0453	0.8728	0.3097	0.029*	
C20	0.2572 (2)	0.90622 (11)	0.31536 (17)	0.0122 (6)	
C21	0.2730 (2)	0.96361 (12)	0.31837 (18)	0.0177 (7)	
H21	0.2423	0.9851	0.2712	0.021*	
C22	0.3328 (3)	0.98882 (13)	0.38964 (19)	0.0215 (7)	
H22	0.3431	1.0276	0.3912	0.026*	
C23	0.3778 (2)	0.95781 (13)	0.45895 (18)	0.0192 (7)	
H23	0.4186	0.9752	0.5080	0.023*	
C24	0.3628 (2)	0.90128 (12)	0.45614 (17)	0.0159 (6)	
H24	0.3940	0.8800	0.5035	0.019*	
C25	0.3028 (2)	0.87543 (12)	0.38493 (17)	0.0148 (6)	
H25	0.2929	0.8367	0.3838	0.018*	
C26	0.5171 (2)	0.91157 (11)	0.23539 (17)	0.0118 (6)	
C27	0.6010 (2)	0.94399 (11)	0.23557 (18)	0.0143 (6)	
H27	0.6070	0.9539	0.1860	0.017*	
C28	0.6755 (2)	0.96181 (12)	0.30703 (18)	0.0180 (7)	
H28	0.7334	0.9828	0.3063	0.022*	
C29	0.6658 (2)	0.94917 (12)	0.37982 (19)	0.0186 (7)	
H29	0.7166	0.9617	0.4289	0.022*	
C30	0.5817 (2)	0.91827 (12)	0.38042 (18)	0.0186 (7)	
H30	0.5743	0.9100	0.4301	0.022*	
C31	0.5079 (2)	0.89923 (12)	0.30910 (17)	0.0145 (6)	
H31	0.4508	0.8777	0.3103	0.017*	
C32	0.4624 (2)	0.90346 (11)	0.06293 (17)	0.0124 (6)	
C33	0.5599 (2)	0.88860 (12)	0.06151 (17)	0.0150 (6)	
H33	0.6063	0.8672	0.1042	0.018*	
C34	0.5894 (2)	0.90472 (12)	−0.00143 (18)	0.0189 (7)	
H34	0.6566	0.8953	−0.0011	0.023*	
C35	0.5212 (2)	0.93456 (12)	−0.06500 (18)	0.0196 (7)	
H35	0.5411	0.9451	−0.1086	0.024*	
C36	0.4244 (2)	0.94896 (12)	−0.06488 (18)	0.0186 (7)	
H36	0.3774	0.9691	−0.1088	0.022*	
C37	0.3950 (2)	0.93417 (11)	−0.00067 (17)	0.0138 (6)	
H37	0.3289	0.9451	−0.0003	0.017*	
C38	0.4531 (2)	0.81004 (11)	0.16173 (16)	0.0111 (6)	
H38A	0.5248	0.8054	0.1623	0.013*	
H38B	0.4546	0.8002	0.2160	0.013*	
C39	0.4238 (2)	0.75692 (11)	0.01009 (17)	0.0116 (6)	
C40	0.5075 (2)	0.72272 (12)	0.01472 (18)	0.0161 (6)	
H40	0.5374	0.6996	0.0602	0.019*	
C41	0.5475 (2)	0.72216 (12)	−0.04627 (18)	0.0184 (7)	
H41	0.6040	0.6984	−0.0427	0.022*	
C42	0.5049 (2)	0.75631 (13)	−0.11252 (18)	0.0191 (7)	
H42	0.5326	0.7563	−0.1541	0.023*	
C43	0.4223 (2)	0.79031 (13)	−0.11797 (18)	0.0194 (7)	
H43	0.3936	0.8139	−0.1632	0.023*	
C44	0.3809 (2)	0.79025 (12)	−0.05756 (17)	0.0157 (6)	
H44	0.3229	0.8131	−0.0624	0.019*	
C45	0.4101 (2)	0.69546 (11)	0.14349 (16)	0.0124 (6)	
C46	0.3359 (2)	0.65358 (12)	0.12815 (18)	0.0157 (6)	
H46	0.2661	0.6604	0.0934	0.019*	
C47	0.3633 (3)	0.60202 (12)	0.16316 (19)	0.0214 (7)	
H47	0.3122	0.5738	0.1521	0.026*	
C48	0.4646 (3)	0.59159 (13)	0.21408 (19)	0.0241 (7)	
H48	0.4834	0.5561	0.2371	0.029*	
C49	0.5384 (3)	0.63302 (13)	0.23138 (19)	0.0245 (7)	
H49	0.6077	0.6261	0.2671	0.029*	
C50	0.5114 (2)	0.68490 (12)	0.19664 (18)	0.0185 (7)	
H50	0.5624	0.7133	0.2092	0.022*	
C51	0.0488 (2)	0.88435 (12)	−0.06356 (16)	0.0137 (6)	
C52	−0.1207 (2)	0.86166 (14)	−0.16977 (19)	0.0248 (7)	
H52A	−0.1557	0.8852	−0.1419	0.030*	
H52B	−0.1606	0.8268	−0.1848	0.030*	
C53	−0.1221 (3)	0.89034 (17)	−0.2447 (2)	0.0373 (9)	
H53	−0.0787	0.9217	−0.2384	0.045*	
C54	−0.1778 (4)	0.8755 (2)	−0.3166 (3)	0.0612 (14)	
H54A	−0.2222	0.8443	−0.3253	0.073*	
H54B	−0.1745	0.8958	−0.3612	0.073*	
C55	0.1704 (3)	0.32731 (15)	0.0731 (2)	0.0354 (9)	
H55A	0.0970	0.3203	0.0645	0.053*	
H55B	0.1846	0.3155	0.0256	0.053*	
H55C	0.1849	0.3668	0.0819	0.053*	
C56	0.2369 (3)	0.29660 (16)	0.1434 (2)	0.0316 (8)	
N3	0.2868 (3)	0.27258 (16)	0.1977 (2)	0.0492 (9)	
N4	−0.0747 (3)	0.95436 (13)	0.43359 (19)	0.0373 (8)	
C58	0.0114 (3)	0.94818 (14)	0.4440 (2)	0.0263 (8)	
C57	0.1216 (3)	0.94051 (16)	0.4589 (2)	0.0344 (9)	
H57A	0.1633	0.9583	0.5098	0.052*	
H57B	0.1383	0.9570	0.4149	0.052*	
H57C	0.1376	0.9010	0.4621	0.052*	
H1A	0.064 (2)	0.9555 (14)	−0.0224 (18)	0.041*	
H1B	−0.0412 (18)	0.9480 (15)	−0.082 (2)	0.041*	
H2A	0.012 (3)	0.8176 (10)	−0.114 (2)	0.041*	

### Atomic displacement parameters (Å^2^)

**Table:**

	*U*^11^	*U*^22^	*U*^33^	*U*^12^	*U*^13^	*U*^23^
Cu1	0.01203 (18)	0.01158 (17)	0.01131 (17)	0.00084 (13)	0.00618 (14)	0.00045 (14)
Cu2	0.01152 (18)	0.01144 (17)	0.01248 (17)	0.00183 (13)	0.00689 (14)	0.00178 (14)
Br1	0.01909 (16)	0.01499 (15)	0.01340 (14)	−0.00064 (12)	0.00622 (12)	−0.00410 (12)
Br2	0.01744 (16)	0.01076 (14)	0.02000 (15)	0.00327 (11)	0.00868 (13)	0.00302 (12)
S1	0.0122 (4)	0.0117 (3)	0.0114 (3)	0.0017 (3)	0.0045 (3)	0.0010 (3)
P1	0.0117 (4)	0.0099 (3)	0.0103 (3)	0.0002 (3)	0.0055 (3)	−0.0005 (3)
P2	0.0132 (4)	0.0090 (3)	0.0111 (3)	0.0006 (3)	0.0072 (3)	−0.0001 (3)
P3	0.0110 (4)	0.0089 (3)	0.0111 (3)	0.0010 (3)	0.0061 (3)	0.0015 (3)
P4	0.0123 (4)	0.0088 (3)	0.0109 (3)	0.0010 (3)	0.0058 (3)	0.0005 (3)
N1	0.0171 (15)	0.0149 (13)	0.0213 (15)	0.0037 (11)	0.0031 (12)	−0.0013 (11)
N2	0.0146 (14)	0.0158 (13)	0.0160 (13)	0.0008 (11)	0.0027 (11)	0.0011 (11)
C1	0.0111 (15)	0.0117 (14)	0.0150 (14)	−0.0024 (11)	0.0082 (12)	0.0001 (12)
C2	0.0210 (17)	0.0159 (15)	0.0152 (15)	−0.0011 (13)	0.0097 (13)	−0.0024 (13)
C3	0.0303 (19)	0.0173 (16)	0.0159 (15)	0.0050 (13)	0.0130 (14)	0.0052 (13)
C4	0.033 (2)	0.0113 (15)	0.0261 (17)	0.0003 (13)	0.0186 (16)	0.0030 (13)
C5	0.0316 (19)	0.0121 (15)	0.0242 (17)	−0.0049 (13)	0.0130 (15)	−0.0064 (14)
C6	0.0220 (17)	0.0159 (15)	0.0137 (15)	0.0000 (13)	0.0068 (13)	0.0010 (13)
C7	0.0127 (15)	0.0090 (14)	0.0176 (15)	−0.0008 (11)	0.0068 (12)	−0.0034 (12)
C8	0.0231 (19)	0.050 (2)	0.0283 (19)	0.0040 (17)	0.0152 (16)	0.0097 (17)
C9	0.0194 (19)	0.047 (2)	0.041 (2)	0.0028 (16)	0.0217 (17)	0.0022 (19)
C10	0.0119 (16)	0.0184 (16)	0.042 (2)	−0.0023 (13)	0.0079 (15)	−0.0130 (15)
C11	0.0218 (19)	0.039 (2)	0.031 (2)	0.0106 (16)	0.0054 (16)	0.0025 (17)
C12	0.0218 (18)	0.0294 (18)	0.0235 (17)	0.0045 (14)	0.0109 (15)	0.0035 (15)
C13	0.0144 (15)	0.0110 (14)	0.0122 (14)	−0.0005 (11)	0.0068 (12)	−0.0014 (12)
C14	0.0146 (15)	0.0078 (13)	0.0157 (14)	−0.0026 (11)	0.0070 (12)	−0.0034 (12)
C15	0.0176 (17)	0.0299 (18)	0.0190 (16)	0.0009 (14)	0.0111 (14)	0.0073 (14)
C16	0.0223 (19)	0.034 (2)	0.0244 (18)	0.0060 (15)	0.0082 (15)	0.0127 (16)
C17	0.0130 (16)	0.0161 (15)	0.0253 (17)	0.0020 (12)	0.0086 (14)	−0.0008 (13)
C18	0.0249 (19)	0.037 (2)	0.0252 (18)	0.0073 (15)	0.0190 (15)	0.0055 (16)
C19	0.0241 (18)	0.035 (2)	0.0179 (16)	0.0112 (15)	0.0121 (14)	0.0116 (15)
C20	0.0119 (15)	0.0138 (14)	0.0131 (14)	−0.0004 (12)	0.0072 (12)	−0.0040 (12)
C21	0.0230 (18)	0.0137 (15)	0.0168 (15)	−0.0006 (12)	0.0077 (14)	−0.0005 (13)
C22	0.0281 (19)	0.0122 (15)	0.0256 (17)	−0.0041 (13)	0.0114 (15)	−0.0033 (14)
C23	0.0193 (17)	0.0229 (17)	0.0181 (16)	−0.0061 (13)	0.0099 (14)	−0.0087 (14)
C24	0.0146 (16)	0.0220 (16)	0.0136 (14)	0.0021 (13)	0.0083 (13)	0.0009 (13)
C25	0.0175 (16)	0.0149 (15)	0.0155 (15)	−0.0001 (12)	0.0102 (13)	−0.0020 (13)
C26	0.0121 (15)	0.0085 (14)	0.0150 (14)	0.0028 (11)	0.0052 (12)	0.0000 (12)
C27	0.0164 (16)	0.0111 (14)	0.0186 (15)	0.0015 (12)	0.0100 (13)	0.0008 (12)
C28	0.0135 (16)	0.0147 (15)	0.0266 (17)	0.0000 (12)	0.0083 (14)	−0.0027 (13)
C29	0.0153 (16)	0.0193 (16)	0.0200 (16)	0.0029 (13)	0.0049 (13)	−0.0065 (13)
C30	0.0191 (17)	0.0223 (16)	0.0171 (16)	0.0037 (13)	0.0097 (14)	0.0002 (13)
C31	0.0140 (15)	0.0159 (15)	0.0153 (15)	−0.0019 (12)	0.0073 (13)	−0.0025 (13)
C32	0.0172 (16)	0.0091 (13)	0.0137 (14)	−0.0026 (12)	0.0088 (12)	−0.0020 (12)
C33	0.0162 (16)	0.0143 (15)	0.0160 (15)	−0.0010 (12)	0.0077 (13)	−0.0003 (12)
C34	0.0201 (17)	0.0170 (15)	0.0265 (17)	−0.0015 (13)	0.0167 (14)	−0.0033 (14)
C35	0.0289 (19)	0.0179 (16)	0.0176 (16)	−0.0057 (13)	0.0151 (14)	−0.0010 (13)
C36	0.0241 (18)	0.0151 (15)	0.0154 (15)	−0.0026 (13)	0.0057 (14)	0.0030 (13)
C37	0.0125 (15)	0.0116 (14)	0.0180 (15)	−0.0036 (11)	0.0066 (13)	0.0009 (12)
C38	0.0126 (15)	0.0111 (14)	0.0110 (14)	0.0003 (11)	0.0060 (12)	0.0006 (12)
C39	0.0112 (15)	0.0113 (14)	0.0134 (14)	−0.0046 (11)	0.0059 (12)	−0.0044 (12)
C40	0.0137 (15)	0.0167 (15)	0.0199 (16)	−0.0006 (12)	0.0085 (13)	−0.0010 (13)
C41	0.0142 (16)	0.0196 (16)	0.0256 (17)	−0.0005 (13)	0.0123 (14)	−0.0055 (14)
C42	0.0207 (17)	0.0247 (17)	0.0171 (15)	−0.0051 (13)	0.0131 (14)	−0.0069 (14)
C43	0.0208 (17)	0.0234 (17)	0.0152 (15)	−0.0012 (13)	0.0081 (13)	0.0007 (13)
C44	0.0184 (17)	0.0132 (15)	0.0168 (15)	0.0019 (12)	0.0081 (13)	−0.0011 (12)
C45	0.0183 (16)	0.0109 (14)	0.0108 (14)	0.0034 (12)	0.0088 (13)	−0.0015 (12)
C46	0.0199 (17)	0.0126 (14)	0.0167 (15)	−0.0004 (12)	0.0091 (13)	−0.0004 (12)
C47	0.0308 (19)	0.0133 (15)	0.0237 (17)	−0.0030 (13)	0.0143 (15)	0.0013 (14)
C48	0.040 (2)	0.0106 (15)	0.0235 (17)	0.0026 (14)	0.0132 (16)	0.0049 (13)
C49	0.0267 (19)	0.0216 (17)	0.0223 (17)	0.0063 (14)	0.0053 (15)	0.0052 (14)
C50	0.0236 (18)	0.0127 (15)	0.0199 (16)	0.0010 (13)	0.0087 (14)	0.0008 (13)
C51	0.0176 (16)	0.0156 (15)	0.0100 (14)	0.0015 (12)	0.0076 (12)	0.0042 (12)
C52	0.0163 (17)	0.0271 (18)	0.0244 (17)	0.0004 (14)	−0.0004 (14)	−0.0026 (15)
C53	0.029 (2)	0.048 (2)	0.026 (2)	0.0088 (17)	−0.0013 (17)	0.0047 (18)
C54	0.063 (3)	0.077 (3)	0.033 (2)	0.026 (3)	0.004 (2)	0.010 (2)
C55	0.026 (2)	0.033 (2)	0.047 (2)	−0.0025 (16)	0.0120 (18)	0.0049 (18)
C56	0.0210 (19)	0.038 (2)	0.039 (2)	−0.0014 (16)	0.0146 (18)	−0.0081 (18)
N3	0.032 (2)	0.073 (3)	0.042 (2)	0.0195 (19)	0.0129 (17)	0.009 (2)
N4	0.030 (2)	0.0415 (19)	0.0396 (19)	0.0002 (15)	0.0113 (15)	−0.0120 (15)
C58	0.028 (2)	0.0248 (18)	0.0266 (18)	0.0004 (15)	0.0112 (16)	−0.0045 (15)
C57	0.031 (2)	0.035 (2)	0.042 (2)	−0.0028 (16)	0.0187 (18)	−0.0069 (18)

### Geometric parameters (Å, º)

**Table:**

Cu1—P1	2.2795 (8)	C23—C24	1.386 (4)
Cu1—P4	2.2803 (8)	C23—H23	0.9500
Cu1—S1	2.3543 (8)	C24—C25	1.389 (4)
Cu1—Br1	2.5126 (4)	C24—H24	0.9500
Cu2—P2	2.2604 (8)	C25—H25	0.9500
Cu2—P3	2.2730 (8)	C26—C27	1.396 (4)
Cu2—S1	2.3520 (8)	C26—C31	1.399 (4)
Cu2—Br2	2.5238 (4)	C27—C28	1.383 (4)
S1—C51	1.731 (3)	C27—H27	0.9500
P1—C7	1.828 (3)	C28—C29	1.388 (4)
P1—C1	1.836 (3)	C28—H28	0.9500
P1—C13	1.844 (3)	C29—C30	1.382 (4)
P2—C14	1.835 (3)	C29—H29	0.9500
P2—C20	1.837 (3)	C30—C31	1.388 (4)
P2—C13	1.850 (3)	C30—H30	0.9500
P3—C32	1.830 (3)	C31—H31	0.9500
P3—C26	1.831 (3)	C32—C37	1.393 (4)
P3—C38	1.853 (3)	C32—C33	1.398 (4)
P4—C45	1.827 (3)	C33—C34	1.383 (4)
P4—C39	1.841 (3)	C33—H33	0.9500
P4—C38	1.848 (3)	C34—C35	1.386 (4)
N1—C51	1.329 (4)	C34—H34	0.9500
N1—H1A	0.842 (18)	C35—C36	1.378 (4)
N1—H1B	0.849 (18)	C35—H35	0.9500
N2—C51	1.317 (4)	C36—C37	1.394 (4)
N2—C52	1.464 (4)	C36—H36	0.9500
N2—H2A	0.853 (18)	C37—H37	0.9500
C1—C2	1.393 (4)	C38—H38A	0.9900
C1—C6	1.399 (4)	C38—H38B	0.9900
C2—C3	1.391 (4)	C39—C44	1.394 (4)
C2—H2	0.9500	C39—C40	1.397 (4)
C3—C4	1.370 (4)	C40—C41	1.387 (4)
C3—H3	0.9500	C40—H40	0.9500
C4—C5	1.387 (4)	C41—C42	1.388 (4)
C4—H4	0.9500	C41—H41	0.9500
C5—C6	1.383 (4)	C42—C43	1.379 (4)
C5—H5	0.9500	C42—H42	0.9500
C6—H6	0.9500	C43—C44	1.391 (4)
C7—C12	1.374 (4)	C43—H43	0.9500
C7—C8	1.393 (4)	C44—H44	0.9500
C8—C9	1.377 (5)	C45—C50	1.395 (4)
C8—H8	0.9500	C45—C46	1.395 (4)
C9—C10	1.368 (5)	C46—C47	1.388 (4)
C9—H9	0.9500	C46—H46	0.9500
C10—C11	1.369 (5)	C47—C48	1.383 (5)
C10—H10	0.9500	C47—H47	0.9500
C11—C12	1.396 (5)	C48—C49	1.381 (5)
C11—H11	0.9500	C48—H48	0.9500
C12—H12	0.9500	C49—C50	1.393 (4)
C13—H13A	0.9900	C49—H49	0.9500
C13—H13B	0.9900	C50—H50	0.9500
C14—C15	1.376 (4)	C52—C53	1.502 (5)
C14—C19	1.387 (4)	C52—H52A	0.9900
C15—C16	1.391 (4)	C52—H52B	0.9900
C15—H15	0.9500	C53—C54	1.284 (5)
C16—C17	1.373 (4)	C53—H53	0.9500
C16—H16	0.9500	C54—H54A	0.9500
C17—C18	1.371 (4)	C54—H54B	0.9500
C17—H17	0.9500	C55—C56	1.460 (5)
C18—C19	1.388 (4)	C55—H55A	0.9800
C18—H18	0.9500	C55—H55B	0.9800
C19—H19	0.9500	C55—H55C	0.9800
C20—C25	1.389 (4)	C56—N3	1.127 (5)
C20—C21	1.408 (4)	N4—C58	1.140 (4)
C21—C22	1.382 (4)	C58—C57	1.453 (5)
C21—H21	0.9500	C57—H57A	0.9800
C22—C23	1.387 (4)	C57—H57B	0.9800
C22—H22	0.9500	C57—H57C	0.9800
			
P1—Cu1—P4	116.49 (3)	C24—C23—H23	120.2
P1—Cu1—S1	114.94 (3)	C22—C23—H23	120.2
P4—Cu1—S1	102.27 (3)	C23—C24—C25	120.8 (3)
P1—Cu1—Br1	110.20 (2)	C23—C24—H24	119.6
P4—Cu1—Br1	110.80 (2)	C25—C24—H24	119.6
S1—Cu1—Br1	100.87 (2)	C20—C25—C24	120.0 (3)
P2—Cu2—P3	119.31 (3)	C20—C25—H25	120.0
P2—Cu2—S1	117.01 (3)	C24—C25—H25	120.0
P3—Cu2—S1	97.45 (3)	C27—C26—C31	118.4 (3)
P2—Cu2—Br2	107.86 (2)	C27—C26—P3	123.8 (2)
P3—Cu2—Br2	108.88 (2)	C31—C26—P3	117.7 (2)
S1—Cu2—Br2	105.21 (2)	C28—C27—C26	120.8 (3)
C51—S1—Cu2	116.91 (10)	C28—C27—H27	119.6
C51—S1—Cu1	116.25 (10)	C26—C27—H27	119.6
Cu2—S1—Cu1	91.74 (3)	C27—C28—C29	120.2 (3)
C7—P1—C1	99.56 (13)	C27—C28—H28	119.9
C7—P1—C13	103.87 (13)	C29—C28—H28	119.9
C1—P1—C13	103.55 (13)	C30—C29—C28	119.5 (3)
C7—P1—Cu1	118.18 (10)	C30—C29—H29	120.2
C1—P1—Cu1	115.38 (9)	C28—C29—H29	120.2
C13—P1—Cu1	114.19 (9)	C29—C30—C31	120.6 (3)
C14—P2—C20	101.87 (12)	C29—C30—H30	119.7
C14—P2—C13	103.65 (13)	C31—C30—H30	119.7
C20—P2—C13	102.91 (13)	C30—C31—C26	120.4 (3)
C14—P2—Cu2	118.58 (9)	C30—C31—H31	119.8
C20—P2—Cu2	111.27 (9)	C26—C31—H31	119.8
C13—P2—Cu2	116.49 (9)	C37—C32—C33	118.8 (3)
C32—P3—C26	104.69 (13)	C37—C32—P3	118.0 (2)
C32—P3—C38	104.10 (13)	C33—C32—P3	123.2 (2)
C26—P3—C38	98.03 (12)	C34—C33—C32	120.6 (3)
C32—P3—Cu2	115.68 (10)	C34—C33—H33	119.7
C26—P3—Cu2	117.19 (9)	C32—C33—H33	119.7
C38—P3—Cu2	114.89 (9)	C33—C34—C35	120.1 (3)
C45—P4—C39	103.19 (13)	C33—C34—H34	119.9
C45—P4—C38	101.10 (13)	C35—C34—H34	119.9
C39—P4—C38	103.25 (12)	C36—C35—C34	119.9 (3)
C45—P4—Cu1	113.34 (10)	C36—C35—H35	120.0
C39—P4—Cu1	117.09 (9)	C34—C35—H35	120.0
C38—P4—Cu1	116.76 (9)	C35—C36—C37	120.4 (3)
C51—N1—H1A	116 (3)	C35—C36—H36	119.8
C51—N1—H1B	123 (3)	C37—C36—H36	119.8
H1A—N1—H1B	120 (4)	C32—C37—C36	120.1 (3)
C51—N2—C52	125.7 (3)	C32—C37—H37	119.9
C51—N2—H2A	114 (3)	C36—C37—H37	119.9
C52—N2—H2A	121 (3)	P4—C38—P3	117.00 (15)
C2—C1—C6	118.7 (3)	P4—C38—H38A	108.0
C2—C1—P1	124.8 (2)	P3—C38—H38A	108.0
C6—C1—P1	116.6 (2)	P4—C38—H38B	108.0
C3—C2—C1	120.3 (3)	P3—C38—H38B	108.0
C3—C2—H2	119.9	H38A—C38—H38B	107.3
C1—C2—H2	119.9	C44—C39—C40	118.5 (3)
C4—C3—C2	120.5 (3)	C44—C39—P4	118.4 (2)
C4—C3—H3	119.7	C40—C39—P4	123.1 (2)
C2—C3—H3	119.7	C41—C40—C39	120.9 (3)
C3—C4—C5	119.9 (3)	C41—C40—H40	119.6
C3—C4—H4	120.1	C39—C40—H40	119.6
C5—C4—H4	120.1	C40—C41—C42	119.9 (3)
C6—C5—C4	120.2 (3)	C40—C41—H41	120.1
C6—C5—H5	119.9	C42—C41—H41	120.1
C4—C5—H5	119.9	C43—C42—C41	120.0 (3)
C5—C6—C1	120.4 (3)	C43—C42—H42	120.0
C5—C6—H6	119.8	C41—C42—H42	120.0
C1—C6—H6	119.8	C42—C43—C44	120.3 (3)
C12—C7—C8	118.5 (3)	C42—C43—H43	119.9
C12—C7—P1	119.4 (2)	C44—C43—H43	119.9
C8—C7—P1	122.0 (2)	C43—C44—C39	120.6 (3)
C9—C8—C7	120.5 (3)	C43—C44—H44	119.7
C9—C8—H8	119.7	C39—C44—H44	119.7
C7—C8—H8	119.7	C50—C45—C46	118.6 (3)
C10—C9—C8	120.4 (3)	C50—C45—P4	124.5 (2)
C10—C9—H9	119.8	C46—C45—P4	116.9 (2)
C8—C9—H9	119.8	C47—C46—C45	120.6 (3)
C9—C10—C11	120.0 (3)	C47—C46—H46	119.7
C9—C10—H10	120.0	C45—C46—H46	119.7
C11—C10—H10	120.0	C48—C47—C46	120.3 (3)
C10—C11—C12	119.8 (3)	C48—C47—H47	119.8
C10—C11—H11	120.1	C46—C47—H47	119.8
C12—C11—H11	120.1	C49—C48—C47	119.8 (3)
C7—C12—C11	120.6 (3)	C49—C48—H48	120.1
C7—C12—H12	119.7	C47—C48—H48	120.1
C11—C12—H12	119.7	C48—C49—C50	120.2 (3)
P1—C13—P2	112.99 (15)	C48—C49—H49	119.9
P1—C13—H13A	109.0	C50—C49—H49	119.9
P2—C13—H13A	109.0	C49—C50—C45	120.5 (3)
P1—C13—H13B	109.0	C49—C50—H50	119.8
P2—C13—H13B	109.0	C45—C50—H50	119.8
H13A—C13—H13B	107.8	N2—C51—N1	121.4 (3)
C15—C14—C19	119.0 (3)	N2—C51—S1	119.8 (2)
C15—C14—P2	118.9 (2)	N1—C51—S1	118.7 (2)
C19—C14—P2	122.1 (2)	N2—C52—C53	112.4 (3)
C14—C15—C16	120.7 (3)	N2—C52—H52A	109.1
C14—C15—H15	119.7	C53—C52—H52A	109.1
C16—C15—H15	119.7	N2—C52—H52B	109.1
C17—C16—C15	120.2 (3)	C53—C52—H52B	109.1
C17—C16—H16	119.9	H52A—C52—H52B	107.9
C15—C16—H16	119.9	C54—C53—C52	124.8 (4)
C18—C17—C16	119.5 (3)	C54—C53—H53	117.6
C18—C17—H17	120.3	C52—C53—H53	117.6
C16—C17—H17	120.3	C53—C54—H54A	120.0
C17—C18—C19	120.8 (3)	C53—C54—H54B	120.0
C17—C18—H18	119.6	H54A—C54—H54B	120.0
C19—C18—H18	119.6	C56—C55—H55A	109.5
C14—C19—C18	120.0 (3)	C56—C55—H55B	109.5
C14—C19—H19	120.0	H55A—C55—H55B	109.5
C18—C19—H19	120.0	C56—C55—H55C	109.5
C25—C20—C21	119.0 (3)	H55A—C55—H55C	109.5
C25—C20—P2	125.2 (2)	H55B—C55—H55C	109.5
C21—C20—P2	115.8 (2)	N3—C56—C55	178.8 (4)
C22—C21—C20	120.4 (3)	N4—C58—C57	178.9 (4)
C22—C21—H21	119.8	C58—C57—H57A	109.5
C20—C21—H21	119.8	C58—C57—H57B	109.5
C21—C22—C23	120.3 (3)	H57A—C57—H57B	109.5
C21—C22—H22	119.9	C58—C57—H57C	109.5
C23—C22—H22	119.9	H57A—C57—H57C	109.5
C24—C23—C22	119.5 (3)	H57B—C57—H57C	109.5
			
C7—P1—C1—C2	−106.2 (3)	C38—P3—C26—C31	−68.3 (2)
C13—P1—C1—C2	0.7 (3)	Cu2—P3—C26—C31	55.1 (2)
Cu1—P1—C1—C2	126.2 (2)	C31—C26—C27—C28	2.4 (4)
C7—P1—C1—C6	73.1 (2)	P3—C26—C27—C28	−177.5 (2)
C13—P1—C1—C6	−180.0 (2)	C26—C27—C28—C29	−2.2 (4)
Cu1—P1—C1—C6	−54.5 (2)	C27—C28—C29—C30	0.5 (4)
C6—C1—C2—C3	1.1 (4)	C28—C29—C30—C31	0.9 (4)
P1—C1—C2—C3	−179.5 (2)	C29—C30—C31—C26	−0.7 (4)
C1—C2—C3—C4	−1.3 (5)	C27—C26—C31—C30	−0.9 (4)
C2—C3—C4—C5	0.2 (5)	P3—C26—C31—C30	178.9 (2)
C3—C4—C5—C6	1.1 (5)	C26—P3—C32—C37	−123.4 (2)
C4—C5—C6—C1	−1.2 (5)	C38—P3—C32—C37	134.2 (2)
C2—C1—C6—C5	0.1 (4)	Cu2—P3—C32—C37	7.2 (3)
P1—C1—C6—C5	−179.3 (2)	C26—P3—C32—C33	57.3 (3)
C1—P1—C7—C12	−126.0 (3)	C38—P3—C32—C33	−45.1 (3)
C13—P1—C7—C12	127.4 (2)	Cu2—P3—C32—C33	−172.1 (2)
Cu1—P1—C7—C12	−0.3 (3)	C37—C32—C33—C34	0.8 (4)
C1—P1—C7—C8	52.1 (3)	P3—C32—C33—C34	−179.9 (2)
C13—P1—C7—C8	−54.6 (3)	C32—C33—C34—C35	−1.8 (4)
Cu1—P1—C7—C8	177.7 (2)	C33—C34—C35—C36	1.0 (4)
C12—C7—C8—C9	0.4 (5)	C34—C35—C36—C37	0.7 (4)
P1—C7—C8—C9	−177.7 (3)	C33—C32—C37—C36	0.9 (4)
C7—C8—C9—C10	0.2 (6)	P3—C32—C37—C36	−178.5 (2)
C8—C9—C10—C11	−0.4 (5)	C35—C36—C37—C32	−1.7 (4)
C9—C10—C11—C12	0.0 (5)	C45—P4—C38—P3	−165.82 (15)
C8—C7—C12—C11	−0.8 (5)	C39—P4—C38—P3	87.62 (17)
P1—C7—C12—C11	177.3 (3)	Cu1—P4—C38—P3	−42.37 (18)
C10—C11—C12—C7	0.6 (5)	C32—P3—C38—P4	−77.63 (18)
C7—P1—C13—P2	−74.17 (17)	C26—P3—C38—P4	174.93 (16)
C1—P1—C13—P2	−177.80 (15)	Cu2—P3—C38—P4	49.89 (17)
Cu1—P1—C13—P2	55.94 (17)	C45—P4—C39—C44	159.7 (2)
C14—P2—C13—P1	79.86 (17)	C38—P4—C39—C44	−95.3 (2)
C20—P2—C13—P1	−174.31 (14)	Cu1—P4—C39—C44	34.5 (2)
Cu2—P2—C13—P1	−52.29 (17)	C45—P4—C39—C40	−20.4 (3)
C20—P2—C14—C15	130.5 (2)	C38—P4—C39—C40	84.5 (3)
C13—P2—C14—C15	−122.8 (2)	Cu1—P4—C39—C40	−145.7 (2)
Cu2—P2—C14—C15	8.1 (3)	C44—C39—C40—C41	0.4 (4)
C20—P2—C14—C19	−51.2 (3)	P4—C39—C40—C41	−179.4 (2)
C13—P2—C14—C19	55.5 (3)	C39—C40—C41—C42	0.7 (4)
Cu2—P2—C14—C19	−173.6 (2)	C40—C41—C42—C43	−0.6 (4)
C19—C14—C15—C16	0.2 (5)	C41—C42—C43—C44	−0.5 (5)
P2—C14—C15—C16	178.6 (3)	C42—C43—C44—C39	1.6 (4)
C14—C15—C16—C17	0.1 (5)	C40—C39—C44—C43	−1.5 (4)
C15—C16—C17—C18	−0.7 (5)	P4—C39—C44—C43	178.3 (2)
C16—C17—C18—C19	1.1 (5)	C39—P4—C45—C50	74.9 (3)
C15—C14—C19—C18	0.1 (5)	C38—P4—C45—C50	−31.7 (3)
P2—C14—C19—C18	−178.2 (3)	Cu1—P4—C45—C50	−157.4 (2)
C17—C18—C19—C14	−0.8 (5)	C39—P4—C45—C46	−103.0 (2)
C14—P2—C20—C25	108.9 (3)	C38—P4—C45—C46	150.4 (2)
C13—P2—C20—C25	1.7 (3)	Cu1—P4—C45—C46	24.6 (2)
Cu2—P2—C20—C25	−123.8 (2)	C50—C45—C46—C47	−2.0 (4)
C14—P2—C20—C21	−74.1 (2)	P4—C45—C46—C47	176.1 (2)
C13—P2—C20—C21	178.7 (2)	C45—C46—C47—C48	0.3 (4)
Cu2—P2—C20—C21	53.2 (2)	C46—C47—C48—C49	1.3 (5)
C25—C20—C21—C22	−0.2 (4)	C47—C48—C49—C50	−1.1 (5)
P2—C20—C21—C22	−177.3 (2)	C48—C49—C50—C45	−0.6 (5)
C20—C21—C22—C23	0.0 (5)	C46—C45—C50—C49	2.1 (4)
C21—C22—C23—C24	0.3 (5)	P4—C45—C50—C49	−175.8 (2)
C22—C23—C24—C25	−0.4 (4)	C52—N2—C51—N1	0.2 (5)
C21—C20—C25—C24	0.1 (4)	C52—N2—C51—S1	−177.1 (2)
P2—C20—C25—C24	177.0 (2)	Cu2—S1—C51—N2	−148.1 (2)
C23—C24—C25—C20	0.2 (4)	Cu1—S1—C51—N2	−41.3 (3)
C32—P3—C26—C27	4.6 (3)	Cu2—S1—C51—N1	34.5 (3)
C38—P3—C26—C27	111.5 (2)	Cu1—S1—C51—N1	141.3 (2)
Cu2—P3—C26—C27	−125.1 (2)	C51—N2—C52—C53	81.3 (4)
C32—P3—C26—C31	−175.2 (2)	N2—C52—C53—C54	129.7 (4)

### Hydrogen-bond geometry (Å, º)

Cg6 is the centroid of the C20–C25 ring.

**Table:**

*D*—H···*A*	*D*—H	H···*A*	*D*···*A*	*D*—H···*A*
C12—H12···N2	0.95	2.56	3.438 (4)	154
C44—H44···S1	0.95	2.87	3.751 (3)	154
C50—H50···N3^i^	0.95	2.57	3.462 (5)	156
C55—H55*C*···N4^ii^	0.98	2.57	3.338 (5)	136
N1—H1*A*···Br2	0.84 (2)	2.74 (2)	3.580 (3)	172 (4)
N1—H1*B*···Br2^iii^	0.85 (2)	2.61 (2)	3.415 (3)	158 (3)
N2—H2*A*···Br1	0.85 (2)	2.63 (2)	3.468 (3)	166 (3)
C31—H31···*Cg*6	0.95	2.97	3.476 (3)	115

Symmetry codes: (i) −*x*+1, *y*+1/2, −*z*+1/2; (ii) −*x*, *y*−1/2, −*z*+1/2; (iii) −*x*, −*y*+2, −*z*.
